# Which learning activities enhance physiotherapy practice? A systematic review protocol of quantitative and qualitative studies

**DOI:** 10.1186/s13643-017-0475-x

**Published:** 2017-04-17

**Authors:** Edmund Leahy, Lucy Chipchase, Felicity Blackstock

**Affiliations:** 1 Physiotherapy, School of Science and Health, Western Sydney University, Campbelltown, New South Wales Australia; 2 School of Allied Health, La Trobe University, Bundoora, Victoria 3086 Australia; 3 Physiotherapy Department, Northern Health, Epping, Victoria 3076 Australia

**Keywords:** Systematic review, Protocol, Physiotherapy, Education, Knowledge translation, Expertise, Professional development

## Abstract

**Background:**

Learning activities are fundamental for the development of expertise in physiotherapy practice. Continuing professional development (CPD) encompasses formal and informal learning activities undertaken by physiotherapists. Identifying the most efficient and effective learning activities is essential to enable the profession to assimilate research findings and improve clinical skills to ensure the most efficacious care for clients. To date, systematic reviews on the effectiveness of CPD provide limited guidance on the most efficacious models of professional development for physiotherapists. The aim of this systematic review is to evaluate which learning activities enhance physiotherapy practice.

**Methods:**

A search of Ovid MEDLINE, EMBASE, Cumulative Index to Nursing and Allied Health Literature (CINAHL), PsycINFO (Psychological Abstracts), PEDro, Cochrane Library, AMED and Educational Resources and Information Center (ERIC) will be completed. Citation searching and reference list searching will be undertaken to locate additional studies. Quantitative and qualitative studies will be included if they examine the impact of learning activities on clinician’s behaviour, attitude, knowledge, beliefs, skills, self-efficacy, work satisfaction and patient outcomes. Risk of bias will be assessed by two independent researchers. Grading of Recommendations Assessment, Development, and Evaluation (GRADE) and Confidence in the Evidence from Reviews of Qualitative research (CERQual) will be used to synthesise results where a meta-analysis is possible. Where a meta-analysis is not possible, a narrative synthesis will be conducted.

**Systematic review registration:**

PROSPERO CRD42016050157

## Background/introduction/rationale

Learning activities are fundamental to the development of expertise in physiotherapy practice, a profession informed by an ever-expanding evidence base [1]. Continuing professional development (CPD) encompasses all learning activities completed by physiotherapists following graduation from entry-level education. CPD is “the maintenance, enhancement and extension of the knowledge, expertise and competence of health professionals throughout their careers” [2]. Researchers and registration bodies further classify CPD into formal and informal learning activities (Table 1) [2–4]. Informal learning activities consist of unstructured activities such as reflection on experience, reading journals and participation in committees [2, 3]. On the other hand, formal learning activities’ categories consist of more structured activities of which there are three main types: (1) participation in activities such as online learning modules, short courses, and courses run by tertiary institutions, (2) presentation or co-ordination of an educational activity, and (3) active participation in research.

**Table 1:** Types of professional development activities compiled from research [3] and physiotherapy registration board documents [2, 4, 28]

Formal learning activities:	
Participant	Attending accredited courses, conferences, seminars, forums, distance learning, further education Completing tertiary courses which lead to a degree or higher degree In-service educational programs^a^ Online learning (interactive discussion and chat rooms) Videoconferencing Peer review^b^ Work-based learning contracts
Teaching	Making presentations Planning or running a course Writing articles
Research	Undertaking research presenting research Courses leading to research degree Writing papers
Informal learning activities:	
Workplace learning	Learning by doing, learning from experience, in-service education^a^, case studies, reflecting on experience, audit of others, discussions with colleagues, peer review^b^, journal clubs, committee membership, shadowing, secondments, clinical supervision, job rotation, journal club, project work, coaching, benchmarking, role expansion, self-assessment questionnaire completion, significant analysis of events, quality assurance activities, being promoted
Professional activities	Professional body involvement, membership of special interest group, mentoring, lecturing^a^, tutoring^a^, examining, branch meetings, organising journal clubs, maintaining or developing specialist skills, being an expert witness, giving presentations^a^, organising courses^a^, supervising research, being national assessor, committee participation, information sharing at meetings
Self-directed	Reading journals or articles, reviewing books or articles, updating knowledge through internet research or TV, keeping a file of progress.

^a^Formal for Physiotherapy Board of Australia [2] and informal for Health and Care Professionals Council [4]

^b^Formal for Physiotherapy Board of New Zealand [28] and informal for Health and Care Professionals Council [4]

Identifying the most efficient and effective learning activities is essential to enable the profession to assimilate research findings and enhance clinical expertise to maximise patient outcomes. Not only would this assist with the design of educational activities, but also managers and clinicians would find this helpful when deciding which learning activities in which to invest their limited time and resources. This is important, as a lack of time has been reported by physiotherapists to be a major barrier to the implementation of evidence-based practice [5–8]. Further, there is a large industry dedicated to formal CPD activities [9]. Indeed, a recent Australian Physiotherapy Association publication advertised 95 short courses by 20 different providers, with some courses running up to 4 weeks and costing over AU$6000 [10]. Understanding which learning activities provide the greatest impact on physiotherapists’ knowledge, expertise and competence as well as the outcomes in their patients is important given the significant costs associated with these courses.

To date, systematic reviews on the effectiveness of CPD provide limited guidance on the most efficacious models for physiotherapists. Reviews focusing on formal learning including courses, audit, feedback and educational outreach report small effect sizes for patient outcomes and professional performance with only moderate effect sizes for changes in knowledge. [11–14]. However, these systematic reviews did not include the full spectrum of learning activities omitting informal CPD [11–13], excluded qualitative research designs and were not specific to the physiotherapy profession [11–14]. These reviews also omitted qualitative research, so do not capture the possible experiences of the learners. As learning can be a highly individualised experience, understanding the possible participant’s interpretations of these experiences is important to fully understand learning activities.

One review included qualitative research designs and compared different models of education; however, that review only examined knowledge translation interventions for allied health practitioners, including but not limited to physiotherapists [15]. This review found equivocal results regarding effectiveness of knowledge translation interventions for professional and patient outcomes [15]. Again, knowledge translation activities included only formal learning activities such as workshops and outreach visits. Further, this review had a limited search strategy focusing on translation of research into practice Thus, a review with a comprehensive search strategy that includes both formal and informal learning activities is required to provide the foundation for determining which learning activities are best suited to improving clinical expertise and patient outcomes. This systematic review will address the question ‘Which learning activities enhance physiotherapists’ practice?’

## Methods/design

### Design

This systematic review protocol is based on the Preferred Reporting Items for Systematic Reviews and Meta-Analyses for Protocols (PRISMA-P) checklist [16] and has been registered on International Prospective Register of Systematic Reviews (PROSPERO, CRD42016050157).

### Study objectives

The aim of this systematic review is to evaluate which learning activities enhance physiotherapy practice. Enhancement of physiotherapy practice will be demonstrated by changes in knowledge, skills, behaviour, self-efficacy, work satisfaction and patient outcomes. As individuals may respond differently to learning activities, the possible experiences, attitudes and beliefs of physiotherapists with regard to the various learning activities will also be explored.

### Eligibility criteria

To be included in this review, studies need to be published in peer reviewed journals or higher degree dissertations. Eligible studies are required to be published in English language. However, there will be no limits on language. Instead, the number of articles excluded due to non-English language will be reported during the selection process. This will give an appreciation of the articles excluded due to language. Studies will not be excluded due to geography or publication date. As this is a new and emerging area of research, both quantitative and qualitative research designs will be included.

#### Participants

Studies will be eligible for inclusion if qualified physiotherapists/physical therapists are participants. Studies where physiotherapists are included with other health professionals (e.g. occupational therapists) will be included if physiotherapy data is reported as a separate subgroup and the educational intervention is considered directly relevant to physiotherapy practice. Studies with participants who are in the process of completing an entry-level physiotherapy/physical therapy degree will be excluded.

#### Interventions

Included studies will need to have participants’ complete formal or informal learning activities that aim to enhance physiotherapy practice. Formal activities include online or face to face short CPD courses, conferences, structured performance feedback, structured electronic reminders (e.g. twitter feeds), structured workplace mentoring and outreach programs (where educator goes to the workplace) and completion of post professional courses towards a degree. Informal activities include talking to colleagues/peers, reflection, committee participation, surfing the internet and reading.

#### Comparisons

Quantitative studies with comparison groups of no educational intervention as a control or comparison groups with a differing educational intervention will be included. Observational pre-post test design studies will be also be included. Qualitative studies will not be required to have a comparison group.

#### Outcomes

Studies will be eligible if they include any outcomes that evaluate change in clinician’s knowledge, skills, behaviour, self-efficacy and work satisfaction. Skills include manual therapy, decision-making, communication and clinical reasoning. The behaviour outcome pertains to how the skills are used in clinical practice. Studies which explore the experiences, attitudes and/or beliefs of the physiotherapist learner will be eligible. Studies will also be eligible if they include patients’ outcomes using reliable and valid measures for that population. Examples of patient outcomes include pain, function and satisfaction with care. Published outcome measure studies using the Consensus-based Standards of health status Measurement Instruments (COSMIN) checklist, will be referred to when determining whether the outcomes used in the studies are reliable and valid [17].

### Information sources

The following electronic databases will be searched from inception to March 2017: Ovid MEDLINE, EMBASE, AMED, Cumulative Index to Nursing and Allied Health Literature (CINAHL), PsycINFO (Psychological Abstracts), PEDro, Cochrane library and Educational Resources and Information Center (ERIC). PubMed will not be searched, as it is the same database as MEDLINE. Citation searching will be completed for included studies using the Science Citation Index Expanded via the Web of Science. Reference lists of included studies will be hand searched for additional included studies.

#### Data management

Data management software that will be used in this review will be Endnote 7 and Microsoft Excel (Office 2013).

### Search strategy

The search strategy used will be developed in consultation with a research librarian employed at the administering institute. Highly sensitive search strategies will be developed by combining index terms and text words relevant to three concepts based on the research questions. The following concepts are: (1) physiotherapists, (2) learning activities and (3) clinician or patient outcomes. Clinician outcomes refer to clinician knowledge, skills, behaviour, knowledge, self-efficacy, work satisfaction, experiences, attitudes, thoughts, feelings and beliefs. An example of full search strategies is provided in Appendix 1.

### Study records

#### Selection process

Studies will be imported from the databases into an Endnote 7 file. Duplicates will be removed, and then the data will be imported into an excel spreadsheet. Data imported will be author, title, journal and year. Two independent researchers (EL, FB) will screen the titles and abstracts for inclusion using the predetermined inclusion criteria. This will be recorded on the excel spreadsheet, which will have columns for included, excluded or unsure. Full-text publications will be sourced for those studies labelled as ‘included’ and ‘unsure’. These will be evaluated for eligibility using the inclusion criteria and recorded on the excel spreadsheet with columns ‘inclusion,’ ‘exclusion,’ and ‘reason for exclusion’. The reason for exclusion column will be broken up further into ‘review’, ‘not English’, ‘pre-qualified students’, ‘data not able to be extracted separate from other health professionals’ and ‘other’. Disagreements at any point during the selection process will be resolved by consensus or a third reviewer (LC).

#### Data collection process

An Excel spreadsheet will be piloted on ten studies for data extraction. Data extraction will be completed by one researcher (EL) and verified by two other researchers (FB, LC).

### Data items

Data extracted will include author, year, study design, sample size, participant characteristics, funding sources, location, setting, context, timeframe, design, results from studies and educational intervention details. Educational intervention details will include whether learning styles and preferences have been considered in the educational design. For quantitative studies, outcome data extracted will include statistically significant results, means and confidence intervals. For qualitative studies, results such as themes will be extracted. Where data are not clearly reported, the original authors will be contacted to clarify missing details.

### Outcomes and prioritisation

Primary outcomes to be collected will be any changes in clinician’s knowledge, skills, behaviour, self-efficacy and work satisfaction. Secondary outcomes to be collected will be valid and reliable measures that evaluate patient change. Qualitative data such as the experiences, thoughts and feelings of clinicians reported as themes and quotes will also be collected related to the primary and secondary outcomes.

### Risk of bias in individual studies

Two independent reviewers (EL, FB) will assess the methodological quality and risk of bias of included studies. Any disagreements will be settled by discussion with a third reviewer (LC) making the final decision if required. If greater than 80% of the quantitative studies are randomised controlled trials, then the PEDro scale [18] will be used. The PEDro scale is a reliable [19] and valid [18] tool for the assessment of clinical trials. While primarily used for the evaluation of randomised controlled trials, it has also been used in a recent systematic review for non-randomised clinical trials to give an appreciation of relative quality of the different study types [20]. This approach allows for a clearer picture of the relative quality of included studies and eliminates the need for using multiple quality scales for different quantitative study types. The PEDro scale consists of 11 items, 10 of which address internal validity components and one which addresses external validity. The scale’s internal validity items cover randomisation, allocation concealment, baseline comparability, blinding, dropouts, intention-to-treat analysis, statistical comparisons as well as reporting of point estimates and variability data. Individual studies are rated as to whether or not they satisfy each of the items. The internal validity items are added to give a final methodological quality score out of 10. PEDro rating results will be communicated in this review via a table which will indicate which items have been satisfied and which have not within each study reporting quantitative data. The final score will also be indicated on this table. If less than 80% of quantitative studies are randomised controlled trials, then a validated and reliable generic risk of bias tool will be used such as the Downs and Black Checklist [21].

The Quality Assessment for Qualitative Research Reports (QAQRR) scale will be used to evaluate risk of bias in qualitative studies [20]. The QAQRR has face validity and has been used in an educationally focussed peer reviewed systematic review [20]. The scale assesses 24 items related to risk of bias in qualitative studies. These items included study design, recruitment methods, sampling strategies, data collection, analysis, disclosure, results and discussion reporting.

Cohen’s Kappa will be calculated to determine the level of inter-rater agreement for risk of bias assessment in both individual qualitative and quantitative studies.

### Data synthesis

Studies will be grouped into qualitative and quantitative studies, which will be analysed separately. Within qualitative and quantitative groups, results for each learning activity will be described. Where appropriate, the qualitative and quantitative will be combined and triangulated in a narrative synthesis.

#### Quantitative data synthesis

Extracted results from quantitative studies will be combined in a meta-analysis where sufficient homogeneity is present. Review Manager 5 software will be used to complete a meta-analysis using a random or fixed effects model, depending on the results from the *I* ^2^ test for heterogeneity [22]. A randomised effects model will be used if *I* ^2^ is greater than 50%. If *I* ^2^ is 50% or less, then a fixed effects model will be used. Heterogeneity will be assessed by considering variability in participant, patient, intervention and outcomes in each study. Where significant heterogeneity is present, study results will not be combined in a formal meta-analysis. Instead, a narrative description of the results will be provided.

Effect sizes will be calculated where possible for each subgroup and communicated in a table to allow for comparison. For continuous outcomes, standardised mean differences with 95% confidence interval (CI) will be reported and analysed. For dichotomous outcomes risk ratio with 95% CI or absolute risk difference with 95% CI will be reported and analysed.

#### Qualitative data synthesis

Where possible, synthesis of data from qualitative studies will be conducted using a thematic analysis approach [23]. Two researchers will independently identify key themes with supporting evidence regarding experiences and thoughts pertaining to professional development courses. Any disagreements will be resolved by consensus or through a third reviewer.

### Meta-biases

To evaluate whether reporting bias is present, reported trials will be compared to their registered protocols, where available, to check if selective reporting of outcomes has occurred.

### Confidence in cumulative evidence

For quantitative data, the Grading of Recommendations Assessment, Development, and Evaluation (GRADE) approach will be used to report on the overall quality of evidence for each outcome [24]. The strength of evidence will be assessed across the domains of type of evidence, risk of bias, consistency, directness, precision and publication bias [25]. Overall quality of the evidence will be rated as either high, moderate, low or very low [25]. Published worksheets will be completed to assist with the GRADE rating of each outcome [26].

For qualitative data, the GRADE-Confidence in the Evidence from Reviews of Qualitative research (CERQual) approach will be used to report the overall confidence of the evidence [27]. The confidence in the evidence will be assessed across the domains of methodological limitations, relevance, coherence and adequacy of data. The overall confidence in the evidence will be rated as either high, moderate, low or very low. A table will be used to summarise key assessment determinations and confidence in the evidence.

## Appendix

### Search strategy example

Medline search

1. physiotherapist*.tw. (5614)
2. (physical adj therapist*).tw. (4660)
3. *Physical Therapists/(645)
4. 1 or 2 or 3 (10339)
5. *educational technology/or *audiovisual aids/or *books, illustrated/or *motion pictures as topic/or *multimedia/or *tape recording/or *videotape recording/or *television/or *microscopy, video/or *videodisc recording/(21306)
6. *Pamphlets/(1561)
7. *Teaching Materials/(2775)
8. 5 or 6 or 7 (25237)
9. *Reminder Systems/(1690)
10. *internet/or *blogging/or *social media/(33694)
11. twitter.tw. (1257)
12. facebook.tw. (1664)
13. e-learning.tw. (1595)
14. *Computer-Assisted Instruction/(7963)
15. 9 or 10 or 11 or 12 or 13 or 14 (44024)
16. (champion* adj change*).tw. (13)
17. (change adj2 agent*).tw. (1396)
18. ((facilitat* or coordinat*) adj2 change*).tw. (4144)
19. 16 or 17 or 18 (5532)
20. mentor$.tw. (10799)
21. educational outreach.tw. (394)
22. *Mentors/(4901)
23. academic detailing.tw. (409)
24. 20 or 21 or 22 or 23 (13214)
25. *Clinical Audit/(429)
26. audit.tw. (27319)
27. *Observation/(1124)
28. observation.tw. (256414)
29. 27 or 28 (256986)
30. *feedback/or *formative feedback/(5441)
31. feedback.tw. (103898)
32. 30 or 31 (105178)
33. 29 and 32 (1893)
34. *"peer review"/or *peer review, health care/(4089)
35. peer review.tw. (7226)
36. 25 or 26 or 33 or 34 or 35 (38120)
37. simulat*.tw. (386052)
38. Standardi* patient*.tw. (2452)
39. Mannequin.tw. (1015)
40. Part task trainer.tw. (20)
41. Virtual reality.tw. (5822)
42. learn*.tw. (283412)
43. Curriculum*.tw. (32461)
44. Teach*.tw. (154046)
45. Feedback*.tw. (105239)
46. Skill*.tw. (148954)
47. 37 or 38 or 39 or 40 or 41 (392108)
48. 42 or 43 or 44 or 45 or 46 (623366)
49. 47 and 48 (25306)
50. professional development.tw. (6263)
51. education.tw. (345369)
52. *education/or *curriculum/or *competency-based education/or *problem-based learning/or *education, distance/or *education, professional/or *education, continuing/or *education, professional, retraining/or *education, graduate/or *education, public health professional/or *inservice training/or *staff development/or *teaching/or *models, educational/or *programmed instruction as topic/or *computer-assisted instruction/or *simulation training/or *patient simulation/(90434)
53. 50 or 51 or 52 (410927)
54. (journal adj club*).tw. (2211)
55. *quality assurance, health care/or *benchmarking/or *clinical audit/or *quality improvement/(41168)
56. *Health Plan Implementation/(2038)
57. (workshop* or seminar* or in-service* or inservice*).tw. (46283)
58. physiotherap* training.tw. (59)
59. physical therap* training.tw. (22)
60. (workshop* or seminar* or in-service* or inservice*).tw. (46283)
61. 54 or 55 or 56 or 57 or 58 or 59 or 60 (91145)
62. *Learning/(32887)
63. learning activit*.tw. (1486)
64. secondment.tw. (83)
65. (reflection or reflective or reflection).tw. (56459)
66. 62 or 63 or 64 or 65 (90215)
67. 8 or 15 or 19 or 24 or 29 or 36 or 49 or 53 or 61 or 66 (930331)
68. *diagnostic self evaluation/or *self-assessment/or *self efficacy/(10658)
69. *Decision Making/(33049)
70. *clinical competence/or *guideline adherence/or *"outcome and process assessment (health care)"/or *"outcome assessment (health care)"/or *"process assessment (health care)"/(81608)
71. *Professional Competence/(10336)
72. *knowledge/(4748)
73. *Health Knowledge, Attitudes, Practice/(46190)
74. ((research or evidence or guideline*) adj3 (implementation or utilization or utilisation or diffusion or translation)).tw. (13861)
75. (increase adj2 implementation).tw. (188)
76. ((predisposing or enabling or reinforcing) adj factor*).tw. (15282)
77. ((support or impede) adj change*).tw. (317)
78. (behavio?r adj2 change*).tw. (18041)
79. (knowledge adj2 (utilization or utilisation or uptake or transfer* or implementation or dissemination or diffusion* or translation)).tw. (4765)
80. (implementation adj2 (program or strategy or strategies)).tw. (5253)
81. (adherence adj3 guidelines).tw. (3432)
82. diagnostic decisio*.tw. (1040)
83. vignett*.tw. (7838)
84. (physiotherap* adj3 quality).tw. (100)
85. *attitude/or *"attitude of health personnel"/or *behavior/or *personal satisfaction/(91820)
86. skill*.tw. (148954)
87. belief*.tw. (63648)
88. 68 or 69 or 70 or 71 or 72 or 73 or 74 or 75 or 76 or 77 or 78 or 79 or 80 or 81 or 82 or 83 or 84 or 85 or 86 or 87 (500801)
89. *"Outcome Assessment (Health Care)"/(23480)
90. *health impact assessment/or *"surveys and questionnaires"/or *health surveys/or *health status indicators/or *self report/(57075)
91. *treatment outcome/(5745)
92. *"Quality of Life"/(64904)
93. *"Recovery of Function"/(9789)
94. functio*.tw. (2952813)
95. impairment*.tw. (260800)
96. participation.tw. (114751)
97. range.tw. (1104948)
98. pain.tw. (490037)
99. (strength or power).tw. (445084)
100. *gait/or *muscle strength/or *hand strength/or *pinch strength/or *pain measurement/or *"range of motion, articular"/or *arthrometry, articular/or *visual analog scale/(41764)
101. *Patient Participation/(11219)
102. adverse event*.tw. (108285)
103. disability.tw. (112551)
104. 89 or 90 or 91 or 92 or 93 or 94 or 95 or 96 or 97 or 98 or 99 or 100 or 101 or 102 or 103 (5007174)
105. 88 or 104 (5374366)
106. 4 and 67 and 105 (1333)

## Abbreviations

CERQual: Confidence in the Evidence from Reviews of Qualitative research
CI: Confidence interval
COSMIN: Consensus-based Standards of health status Measurement Instruments
CPD: Continuing professional development
GRADE: Grading of Recommendations Assessment, Development, and Evaluation
PRISMA-P: Preferred Reporting Items for Systematic Reviews and Meta-Analyses for Protocols
QAQRR: Quality Assessment for Qualitative Research Reports
